# Misfit Layer Compounds as Ultratunable Field Effect Transistors: From Charge Transfer Control to Emergent Superconductivity

**DOI:** 10.1021/acs.nanolett.3c01860

**Published:** 2023-07-07

**Authors:** Ludovica Zullo, Giovanni Marini, Tristan Cren, Matteo Calandra

**Affiliations:** † Department of Physics, University of Trento, Via Sommarive 14, 38123 Povo, Italy; ‡ Sorbonne Université, CNRS, Institut des Nanosciences de Paris, UMR7588, F-75252 Paris, France; ¶ Graphene Laboratories, Fondazione Istituto Italiano di Tecnologia, Via Morego, I-16163 Genova, Italy

**Keywords:** 2D materials, heterostructures, doping, superconductivity

## Abstract

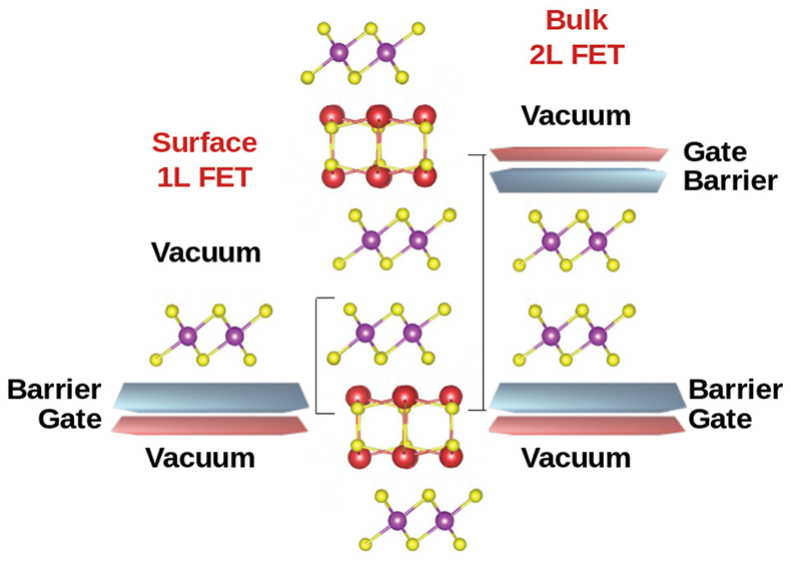

Misfit layer compounds are heterostructures composed of rocksalt units stacked with few-layer transition metal dichalcogenides. They host Ising superconductivity, charge density waves, and good thermoelectricity. The design of misfits’ emergent properties is, however, hindered by the lack of a global understanding of the electronic transfer among the constituents. Here, by performing first-principles calculations, we unveil the mechanism controlling the charge transfer and demonstrate that rocksalt units are always donor and dichalcogenides acceptors. We show that misfits behave as a periodic arrangement of ultratunable field effect transistors where a charging as large as ≈6 × 10^14^ e^–^ cm^–2^ can be reached and controlled efficiently by the La–Pb alloying in the rocksalt. Finally, we identify a strategy to design emergent superconductivity and demonstrate its applicability in (LaSe)_1.27_(SnSe_2_)_2_. Our work paves the way to the design synthesis of misfit compounds with tailored physical properties.

The capability of inducing a controlled and tunable number of carriers in few layer systems has been pivotal for the success of 2D materials.^1^ However, in metallic few layer 2D dichalcogenides such as NbSe_2_, the largest carrier doping that can be achieved via field effect gating is on the order of *n*_*e*_ ≈ 3 × 10^14^ e^–^ cm^–2^,^2^ corresponding to a Fermi level shift on the order of 0.1 eV, too small to drastically change the physical properties.

Recently,^3^ it has been shown that overcoming this limit is possible in the misfit layer compound (MLC) (LaSe)_1.14_(NbSe_2_)_2_, a heterostructure composed of periodically alternating rocksalt monochalcogenide units (RS) and few layer transition metal dichalcogenides (TMDs).^4,5^ In this system, a massive electron transfer from the LaSe RS to the NbSe_2_ TMD occurs, leading to a rigid Fermi level shift as large as +0.55 eV. It is, however, unclear if the electron doping in misfits can be in some way controlled by any physical parameter and, more importantly, how general this mechanism to dope few layer TMDs is.

MLCs have been known for a long time and their structures as a function of the RS and TMD composition have been thoroughly investigated.^4,5^ However, the exploration of physical properties such as Ising superconductivity,^6−11^ charge density waves (CDW),^12−16^ or topological effects^17^ is quite recent. The research in the field has led to remarkable results, but it has mostly proceeded by isolated discoveries and trial and error chemical synthesis, while general rules to understand what happens when assembling different RS units and TMDs are missing. The need for a global picture becomes evident when considering that (i) many ternary alloys composed of monochalcogenides can be assembled with practically any few layer dichalchogenide and (ii) the thickness of the dichalcogenide layers can be chosen at will. This makes a lot of possible combinations and leads to many unanswered questions. For example, how does the charge transfer occur in these structures? Are the TMD layers acceptors or donors? How can the charge transfer be tuned? To what extent is the electronic structure of the TMD is affected when inserted in the heterostructure? Most important, what are the emergent properties of the misfit, i.e., properties of the MLC that are absent in the pristine constituents? How can we design misfit properties from the knowledge of their building blocks?

In this work, we answer these questions by performing extensive first-principles electronic structure calculations of MLCs. We identify the fundamental mechanism ruling charge transfer and demonstrate how the charge injection into the TMD layers can be efficiently controlled by chemical alloying in the rocksalt unit. Most importantly, we show that superconductivity can emerge in MLCs formed by assembling nonsuperconducting RS and TMDs. Finally, we demonstrate that misfit layer compounds can be assimilated to ultratunable field effect transistor with an unequaled charging of the TMD layers. Our work paves the way to extensive experimental synthesis and development of these promising systems.

The chemical formula of MLCs is (RQ)_1+δ_(TX_2_)_*m*_, where (TX_2_)_*m*_ is a *m*-layer TMD and RQ is a rocksalt monochalcogenide unit (often referred to as Q-layer).^4,5^ Ternary alloys of two monochalcogenides within a single RS Q-layer (e.g., La_*x*_Sr_1–*x*_S) have also been synthesized^18^ leading to MLCs having chemical formulas of the kind (R_*x*_M_1–*x*_Q)_1+δ_(TX_2_)_*m*_. As a prototypical example of the MLCs crystal structure, we consider (LaSe)_1.18_(TiSe_2_)_2_, shown in Figure 1. This system is interesting as bulk TiSe_2_ displays a rich phase diagram showing the coexistence of superconductivity and charge density wave. Each TiSe_2_ and LaSe sublattice has its own set of cell parameters. Compared to bulk 1T-TiSe_2_, the lattice of the TiSe_2_ bilayer in the MLC is not perfectly hexagonal as it is slightly expanded along one direction and is described by a centered orthorhombic cell with in-plane lattice vectors **a**_1_ ≈ 3.6 Å and **b**_1_ ≈ 6 Å. The LaSe sublattice has orthorhombic symmetry but with similar in-plane lattice parameters, **a**_2_ ≈ **b**_2_ ≈ 6 Å. Both systems have the same **b** vectors (**b**_1_ ≈ **b**_2_) so that the material is commensurate along this direction. The ratio between |**a**_1_| and |**a**_2_| is an irrational number (see Figures S1 and S2 in the Supporting Information) making the MLC incommensurate in the **a** direction. The mismatch ratio *a*_2_/*a*_1_ = *x*/*y* is usually in the range ∼1.6–1.8 and sets the parameter δ in the chemical formula through the relation 1 + δ = 2 × (*a*_1_/*a*_2_). In this work, we adopt the convention of using the value of δ as obtained from the lattice parameters **a**_1_ and **a**_2_ of the pristine RS and TMD before assembling them in a MLC structure, as reported in the tables in Figures S1 and S2 in the Supporting Information. The commensurate approximant of each MLCs is reported in Figure S3 in the Supporting Information.

**Figure 1:**
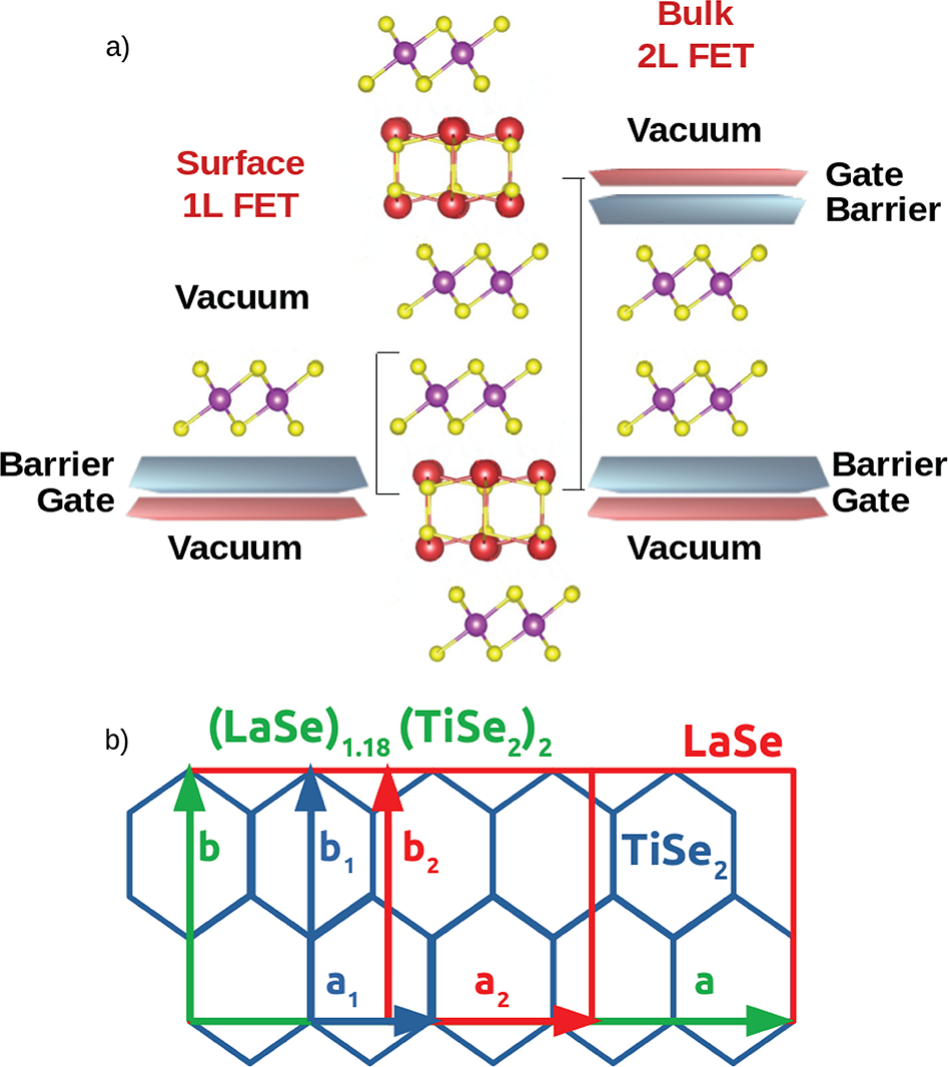
Bulk structure (a) and sketch of the unit cell (b) of (LaSe)_1.18_(TiSe_2_)_2_. The field-effect modeling scheme is depicted for the case of the most common TiSe_2_ terminated surface (left) and for bulk TiSe_2_ (right). The vectors (**a**, **b**) are those of the MLC while the vectors of a TiSe_2_ monolayer are (**a**_1_, **b**_1_) and those of a LaSe unit (**a**_2_, **b**_2_).

The RS layers have strong intralayer bonding. A strong bonding also forms among the RS and TMD layers. On the contrary, van der Waals bonding occurs among the closer TMD layers. After cleavage, for *m* > 1, the surface of the sample is a perfect TMD layer (a single layer in the *m* = 2 case considered in this work^3^). In the *m* = 1 case, i.e., a single layer TMD sandwiched among RS Q-layers, the bonding along the *z* axis is always strong. The cleavage occurs between the RS and TMD bonding and the surface is still a TMD single layer; however, it is often less clean.^6,9^ In all cases, there is a substantial experimental evidence^3^ that ARPES and STS/STM measurements mostly sample the terminating TMD layer without accessing the bulk of the structure. On the contrary, Raman, transport, and superconducting measurements probe bulk properties of the crystal.

In order to gain insight into the charge transfer among the RS and TMD layers in the MLC, we perform extensive calculations of the work functions of 8 isolated rocksalt Q-layers and 12 isolated TMDs single layers. The choice of considering TMD single layers is motivated by (i) the fact that we consider MLC with *m* = 2 having a single layer TMD as the terminating surface and (ii) by the fact that the work functions of bilayers TMDs is fairly close to the one of single layers.^19^ Thus, we expect that our results will also hold for the surface and the bulk and for the *m* = 1 case. Calculations are performed with the quantum ESPRESSO(20) package and we use the PBE exchange and correlation functional^21^ (see the Supporting Information for more technical details). It is worth noting that the GW approximation leads to work functions that are, at most, 0.5 eV larger than the PBE case in TMDs. Moreover, we verified on some rocksalt bilayers (LaSe, PbSe) that HSE06^22^ does not change quantitatively and qualitatively the picture. Results are listed in Figure 2.

**Figure 2:**
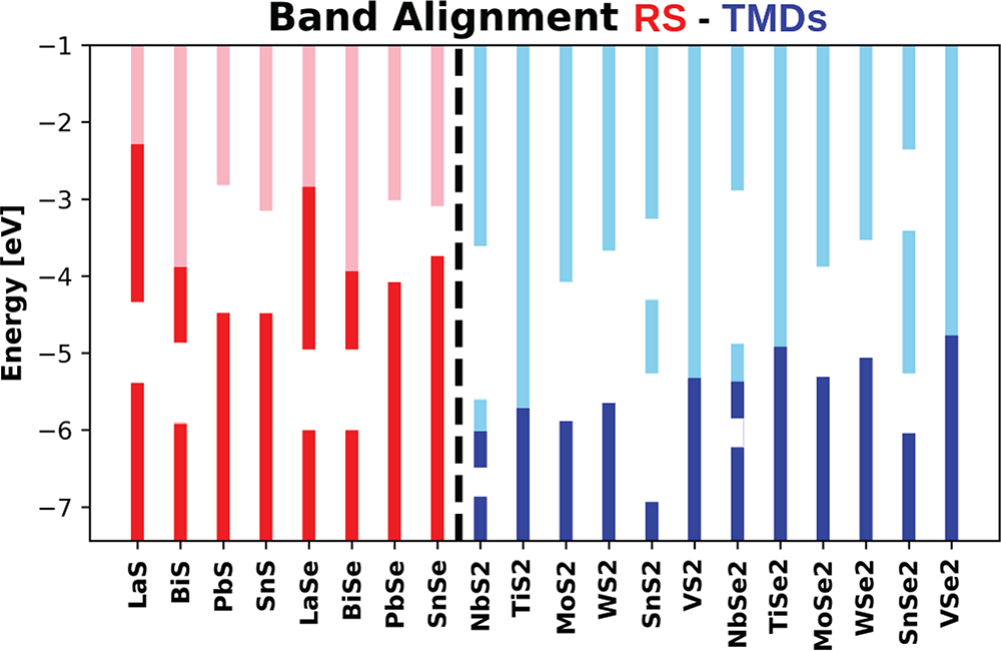
Band alignement of isolated bilayer rocksalt (red) and single layer transition metal dichalcogenides (blue). Dark (light) bars represent the position of the *E*_F_/valence band maximum (*E*_F_/conduction band minimum) for metals/insulators. White spaces in the bars stand for gaps in the single particle spectrum. The energy zero is set to the vacuum level. Electronic structures of the isolated rocksalt bilayers can be found in Figures S9 and S10 in the Supporting Information.

The key quantities ruling the charge transfer in these systems are the work function difference among RS and TMDs and the consequent band alignment, the lattice mismatching ratio *a*_2_/*a*_1_ and, finally, the degree of hybridization when the two subsystems are in contact. As shown in Figure 2, the TMDs globally possess work functions substantially larger than those of the RS compounds. As the work function is the energy required to transfer an electron from the Fermi level to the vacuum level, RS is always the donor and TMDs always acceptors. The net amount of charge transfer depends, however, not only on the work function difference but also on the mutual concentration of the RS and TMD that is related to the mismatching ratio. To explain this more clearly, each RS can transfer a given amount of charge to the TMD layer, if the mismatching ratio is close to one. However, if the mismatching ratio increases, the relative concentration of RS atoms per TMD cell decreases and so does the charge transfer. By looking at Figure 3S in the Supporting Information, it is clear that the mismatching ratio varies mostly due to the change in the TMD lattice parameter.

In order to demonstrate this global picture, we perform explicit calculations for several misfit surfaces terminated by a single layer NbSe_2_ but having different RS units as building blocks and sharing comparable mismatching ratios very close to 7/4 (these compounds belong to the ninth column in Figure 3S in the Supporting Information). As can be seen in Figure 3, the behavior of the Misfit NbSe_2_ 7/4 series is almost completely characterized by the work function differences. Indeed as *W*(LaSe) < *W*(SnSe) < *W*(PbSe), the charge transfer decreases by progressively decreasing the difference *W*(NbSe_2_) – *W*(RS), as expected. The work function of BiSe is slightly larger than the one of SnSe; however, BiSe seems to transfer few more electrons than SnSe. The differences are due to fine details in the electronic-states hybridization.

**Figure 3:**
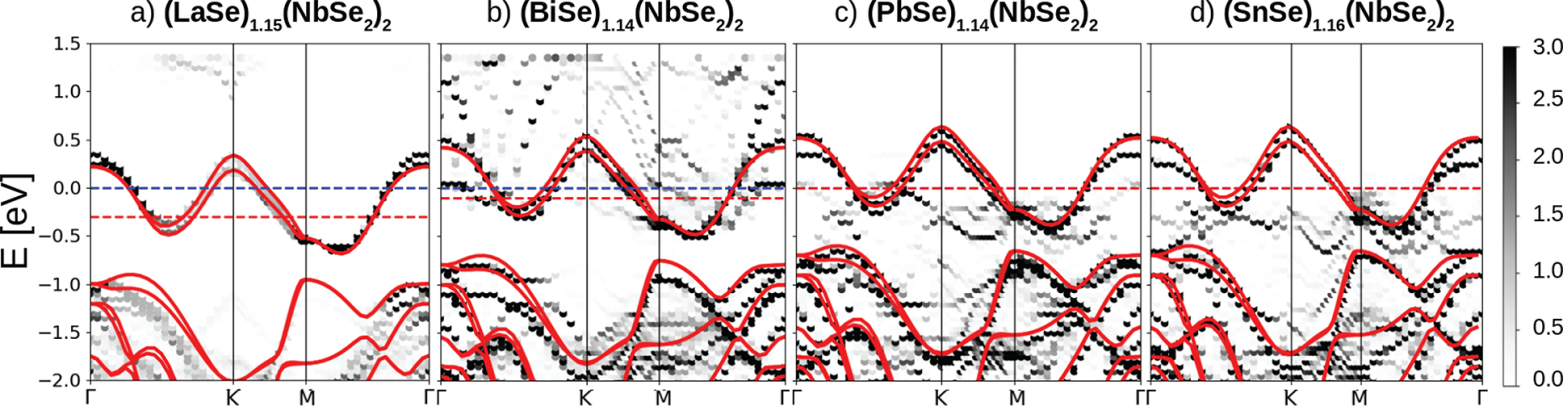
Band unfolding onto the NbSe_2_ single layer Brillouin zone for the NbSe_2_ misfit series for different rocksalt Q-layers having a comparable mismatching ratio close to 7/4. (a) (LaSe)_1.15_ (NbSe_2_)_2_, (b) (BiSe)_1.14_ (NbSe_2_)_2_, (c) (PbSe)_1.14_ (NbSe_2_)_2_, and (d) (SnSe)_1.16_ (NbSe_2_)_2_. The band structure for the isolated single layer NbSe_2_ (red line) is superimposed and aligned to the Nb d-band in the misfit. The blue dashed line corresponds to the Fermi level *E*_F_ of the misfit compound, while the red one corresponds to the Fermi level of the isolated NbSe_2_ layer. In the last two panels the dashed red line is superimposed to the dashed blue one.

Finally, we point out that the NbSe_2_ electronic structure in going from (PbSe)_1.14_(NbSe_2_)_2_ to (LaSe)_1.15_(NbSe_2_)_2_ is n-doped rigidly; i.e., the charge transfer simply induces a Fermi level upshift. From this analysis two questions arise: how general is this rigid doping effect and how can it be used to effectively tune the doping ? We now show that it is possible to engineer the misfit in such a way that the doping level is rigidly adjustable through appropriate alloying of the RS Q-layer.

For this reason, we consider MLCs having the following stoichiometry (La_*x*_Pb_1–*x*_Se)_1.18_(TiSe_2_) as a function of *x*. We point out that similar substitutions (La ↔ Sr) have already been achieved in sulfur-based MLC.^18^ A comparison between this system and the previous results for the NbSe_2_ series allows us to draw conclusions that are less dependent on the chosen TMD.

From the previous reasoning and from Figure 2, we expect that the La concentration (*x*) allows tuning of the carrier concentration in the TiSe_2_ layers with *x* = 1 (*x* = 0) corresponding to the highest (lowest) n-doping. In Figure 4 we show the calculated band structure of the full (La_*x*_Pb_1–*x*_Se)_1.18_(TiSe_2_)_2_ misfit for *x* = 1.0, 0.34, and 0.0 compared with that of an isolated single layer. As can be seen, by increasing *x*, the doping is increased. Most importantly, the Ti d-band displays no deformation upon doping. At the highest doping level (*x* = 1, corresponding to a charge transfer of 0.53 electrons per Ti, which is *n*_*e*_ ∼ 5 × 10^14^ e^–^ cm^–2^) two parabolic La bands cross the Fermi level along the ΓK direction. These bands disappear by decreasing *x* (see the Supporting Information for the calculation at additional values of *x*). Remarkably, the electronic structure of (PbSe)_1.18_(TiSe_2_)_2_ is almost indistinguishable from that of the isolated TiSe_2_ layer.

**Figure 4:**
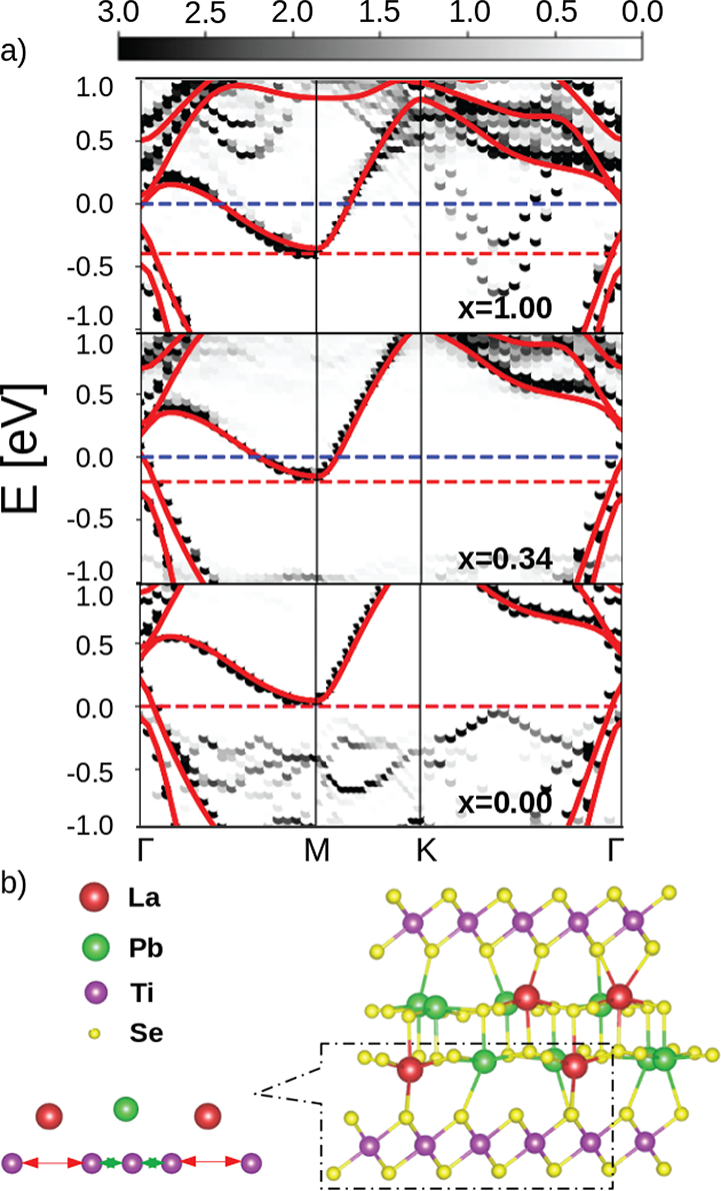
(a) Band unfolding onto the single layer TiSe_2_ Brillouin zone of the misfit compound (La_*x*_Pb_1–*x*_Se)_1.18_(TiSe_2_)_2_ for *x* = 1.0, 0.34, 0.0. The band structure for the isolated single layer TiSe_2_ (red line) is superimposed and aligned with the bottom of the Ti d-band in the misfit. The blue dashed line corresponds to the Fermi level *E*_F_ of the misfit compound, while the red one corresponds to the Fermi level of the isolated TiSe_2_ layer (in the lowest panel, they coincide). (b) Lattice deformation of the TiSe_2_ layers generated by the partial substitution of Pb atoms in (La_*x*_Pb_1–*x*_Se)_1.18_(TiSe_2_)_2_. The magnified portion shows a bond length alternation in the TiSe_2_ lattice with two different distances, *d*_1_ (red) and *d*_2_ (green).

Despite this similarity in the electronic structure, we find that (PbSe)_1.18_(TiSe_2_)_2_ does not display a 2 × 2 CDW as it happens in the case of the supported TiSe_2_ single layer.^23−25^ This result is in agreement with resistivity data on this MLC^7^ where no CDW was detected. We attribute the suppression of the CDW to the strong bonding between TiSe_2_ and the RS Q-layer. We find that in (La_*x*_Pb_1–*x*_Se)_1.18_(TiSe_2_)_2_, for *x* ≠ 0, 1, the Ti–Ti distances are modulated by the presence of Pb atoms in the host LaSe lattice (i.e., the Ti–Ti distance becomes shorter if the Ti atoms are close to a Pb atom). The reason is mostly sterical as the La atomic radius is larger than the one of Pb; therefore, Pb atoms are more strongly bounded to the RS layer, and a consequent deformation of the LaSe rocksalt host occurs (as shown in Figure 4(b)) followed by a modulation of the Ti–Ti distances. We verified that even starting from 2 × 2 distorted TiSe_2_ layers in the misfit, the structural optimization suppresses the CDW and leads to other distortion patterns that essentially follow the Pb atoms superstructure. Our analysis shows that altering the chemical composition of the rocksalt has a double effect: on the one hand, it allows us to precisely tune the rigid doping of the TMD; on the other hand, it suppresses the 2 × 2 CDW of the TiSe_2_ bilayer and introduces an additional modulation related to the alternation of La and Pb.

After achieving complete knowledge of the charge transfer in MLC, we now demonstrate how to design a misfit superconductor starting from its constituents. In particular, we show that nonsuperconducting pristine RS and TMD compounds can lead to a superconductor via charge transfer control (emergent superconductivity).

We consider the layered indirect gap semiconductor 1TSnSe_2_ that can be exfoliated and synthesized in single layer form.^26^ The electronic structure of single-layer SnSe_2_ is shown in Figure 5 (red line). The conduction band is formed by an isolated band with a Van Hove singularity point at K. A maximum in the density of states occurs at the energy corresponding to the band flattening. If the Fermi level is tuned at the inflection point, this would be beneficial for superconductivity. However, this involves a ≈1.4 eV Fermi level shift corresponding to a charge transfer of 0.77 electrons (≈6 × 10^14^ e^–^ cm^–2^), unreachable even in an ionic-liquid-based field effect transistor. However, as previously shown, this electron doping level could be reached in misfit (La_*x*_Pb_1–*x*_Se)_1.27_(SnSe_2_)_2_. In order to confirm this hypothesis, we perform first-principles calculations for this MLC as a function of *x* (see Figure 8 in the Supporting Information). We find that the La–Pb alloying allows perfect control of the doping level due to the large work function difference between LaSe and SnSe_2_ and an insulator-to-metal transition occurs in SnSe_2_. At *x* = 1 the Fermi level perfectly matches the inflection point. It is worth noting that at this high La concentration, some LaSe bands cross the Fermi level close to the K point and along ΓK; however, their contribution to the total density of states is marginal. In Figure 5 we also compare the MLC surface electronic structure with the one of an isolated layer (red line). There is substantial band distortion with respect to the isolated single layer. A better description of the surface electronic structure is obtained by replacing the LaSe layer with a uniformly positive charged potential barrier, as in a single gate field effect transistor setup by using the method developed in ref (27). The electronic structure of an isolated SnSe_2_ layer under this approximation is the green line in Figure 5, in perfect agreement with the complete calculation of the MLC surface electronic structure both for what concerns the band bending at the Fermi level (some deviations are seen in the empty states close to the zone center) and for the position of the valence band top. We attribute the band bending occurring at the K high-symmetry point to a modification of the intralayer spacing between Sn and Se in SnSe_2_ due to the charging of the monolayer (a table with intralayer spacing comparisons can be found in Figure 7 of the Supporting Information).

**Figure 5:**
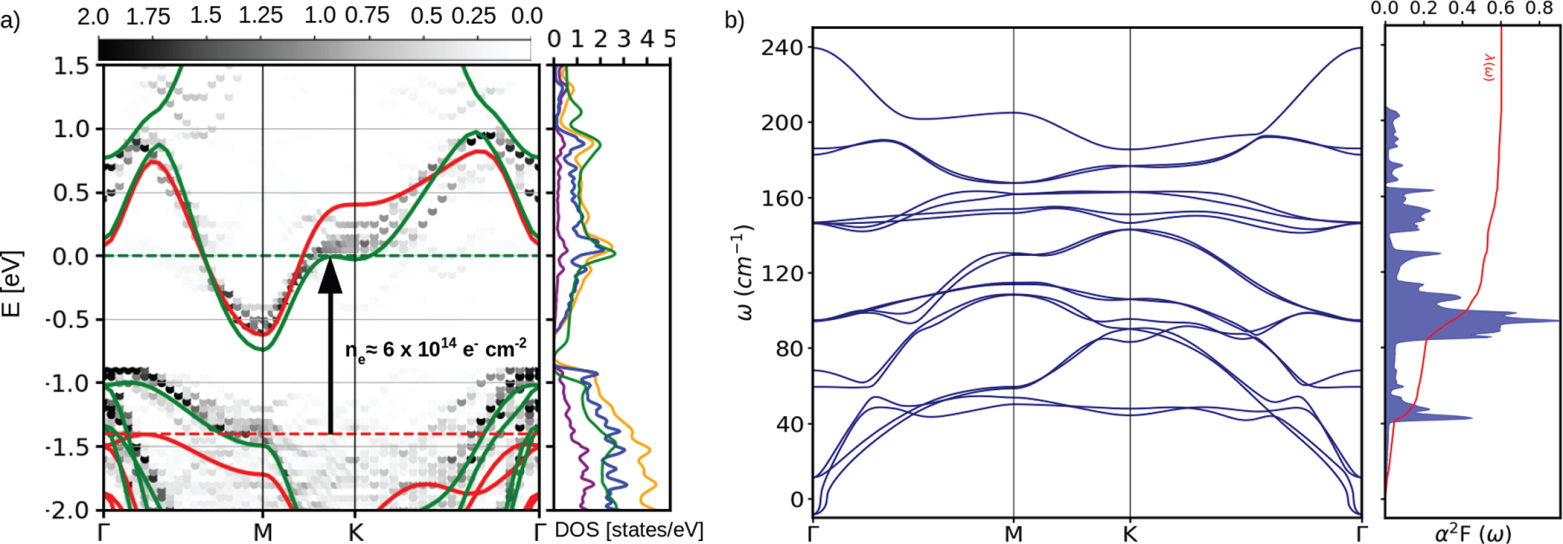
(a) Band unfolding of (LaSe)_1.27_(SnSe_2_)_2_ misfit supercell onto the hexagonal primitive Brillouin Zone (BZ) of single layer SnSe_2_ (the zero energy is set to the Fermi level, dashed green line). The superimposed solid lines are the band structure of an isolated single layer SnSe_2_ (red) and of a single layer SnSe_2_ doped in a single FET setup as in (LaSe)_1.27_(SnSe_2_)_2_ by 0.7 electrons per Sn atoms (green), respectively. Darker regions in the colormap represent the most relevant projection of the misfit eigenvalues of the band structure in the SnSe_2_ first BZ (band unfolding). In the adjacent panel, we plot the total DOS per SnSe_2_ formula unit of (LaSe)_1.27_(SnSe_2_)_2_ (yellow) and the projected density of states over atomic orbitals of the LaSe layers (purple) and of the SnSe_2_ layers (blue), respectively. The green line is the DOS of a single layer of SnSe_2_ doped in a single FET setup of 0.7 electrons per Sn atoms. (b) Dynamical properties and electron–phonon coupling of (LaSe)_1.27_(SnSe_2_)_2_ modeled by a bilayer SnSe_2_ in a double FET setup. The phonon dispersion is shown in the first panel, while in the adjacent panel, the Eliashberg function α^2^*F*(ω) (filled blue curve) and the total electron–phonon coupling λ(ω) (red) are depicted.

This result shows that it is possible via Pb/La alloying in the RS layers to set the Fermi level at the Van Hove singularity. Furthermore, it shows that the LaSe Q-layer can be assimilated to a capacitor plate in a Field Effect Transistor (FET) (Figure 1(a)). This remains true even for the SnSe_2_ bilayers in the bulk of the sample; i.e., the full MLC can be assimilated to several field-effect transistors stacked periodically along the *z*-axis of the MLC, as shown in Figure 1.

As superconductivity is a bulk property, we must simulate the complete 3D crystal. The calculation of the vibrational properties and electron–phonon coupling for the complete MLC is, however, a very cumbersome task due to the large number of atoms. We then proceed differently; namely, we consider a SnSe_2_ bilayer in a field effect configuration as in Figure 1 with a +0.7 charge on each of the two plates (double gate configuration). In order to prevent the ions from moving too close to the gate electrodes, a potential barrier is placed before the gates, and the total charge of the system is maintained equal to zero.^27^ Additional details on these calculations can be found in the Supporting Information. We have verified that this approach gives geometries for the SnSe_2_ bilayer, in excellent agreement with the complete MLC structural optimization. Furthermore, the electronic density of states of the MLC and that of the monolayer in double gate configuration are practically indistinguishable, as shown in Figure 5.

We then calculate the phonon dispersion (ω_**q**ν_) and the electron–phonon coupling λ_**q**ν_ for each mode ν of phonon crystal momentum **q** in double gate geometry. From these quantities we obtain the Eliashberg function α^2^*F*(ω) =  and the average electron–phonon coupling λ =  = 0.6, *N*_*q*_ = 96 × 96 being the number of points in the phonon momentum grid used to calculate the average (see the Supporting Information). These quantities are plotted in Figure 5(b). Approximately 30% of the coupling arises from the Einstein optical modes at ≈45–50 cm^–1^, while the rest of the coupling is uniformly distributed throughout the other modes. The phonon density of states (not shown) is very similar to that of the Eliashberg function.

We calculate the superconducting critical temperature by solving the anisotropic Migdal–Eliashberg equations,^28^ as implemented in the EPIq software,^29−31^ and by assuming μ* = 0.1, obtaining *T*_*c*_ = 3.5 K (see the Supporting Information for details on Migdal–Eliashberg calculations). This result matches well with the *T*_*c*_ = 4.8 K detected in ultrathin Li-intercalated SnSe_2_ via field effect gating and demonstrates that superconductivity can emerge in MLC from pristine components that are not superconducting.

In conclusion, by performing extensive first-principles electronic structure calculations on misfit layer compounds, we unveiled the mechanism ruling charge transfer in these systems. In particular, due to their large work functions, we showed that TMDs are always acceptors, while rocksalts are always donors. The electron density that can be injected in the TMD layers can be as high as 6 × 10^14^ e^–^ cm^–2^, sensibly larger than in ordinary field-effect transistors.

We have shown that the charging of the TMD layers can be efficiently controlled via La ↔ Pb substitution. Most interesting, by replacing each RS Q-layer with a charged plate and a barrier, we have shown that the surface of the MLC behaves as a single gated field-effect transistor while the bulk can be seen as a periodic arrangement of a double-gated field effect transistor.

Finally and most importantly, we have shown that from the knowledge of the RS and TMD constituents, it is possible to infer the amount of charge transfer to the TMD layers in the MLC and to predict the physical properties of the heterostructure. As a practical demonstration, we showed that emergent superconductivity occurs in (LaSe)_1.27_(SnSe_2_)_2_ via a 1.4 eV Fermi level shift induced by the presence RS Q-layers in the misfit. The methodology developed in this work paves the way for the synthesis and design of misfit compounds with tailored physical properties.

## Supporting Information Available

Geometrical details of MLCs, technical details, band alignment calculation, band unfolding method applied to (La_*x*_Pb_1–*x*_Se)_1.18_(TiSe_2_)_2_, doping-induced superconductivity, and electronic structure of isolated rocksalt bilayers (PDF)
