# Supplementary material for: Misfit Layer Compounds as Ultratunable Field Effect Transistors: From Charge Transfer Control to Emergent Superconductivity
Source: Nano Lett. 2023 Jul 7;23(14):6658–63. doi: 10.1021/acs.nanolett.3c01860 (PMC10375578; doi:10.1021/acs.nanolett.3c01860)
Supplement: Supplementary file 1 — nl3c01860_si_001.pdf [file nl3c01860_si_001.pdf]

Supporting Information for "Misfit layer compounds as ultra-tunable field effect transistors: from charge transfer control to emergent superconductivity"

Ludovica Zullo,*,†,‡ Giovanni Marini,¶ Tristan Cren,‡ and Matteo Calandra*,†,‡,¶

†*Department of Physics, University of Trento, Via Sommarive 14, 38123 Povo, Italy*

‡*Sorbonne Université, CNRS, Institut des Nanosciences de Paris, UMR7588, F-75252 Paris, France*

¶*Graphene Labs, Fondazione Istituto Italiano di Tecnologia, Via Morego, I-16163 Genova, Italy*

E-mail: ludovica.zullo@unitn.it; m.calandrabuonaura@unitn.it

I. Geometrical Details of MLCs

In Fig. S1 and S2 we report the exact mismatch ratios a_2/a_1 and exact misfits stechiometry $1+\delta = 2\times(a_1/a_2)$ respectively, starting from the experimental lattice parameters of each isolated considered rocksalts and TMDs. In order to build commensurate misfit supercells, from these analysis we extract the approximant used in the calculations (Fig. S3). We now illustrate how the misfit supercell is built, specifically considering the cases of single layer $TiSe_2$ and $SnSe_2$, both found in the 1T phase. For both cases, we start by considering the orthorhombic cell and optimize their lattice parameters. Lattice parameters in the

a_2 (Å) \ a_1 (Å)		3.15	3.15	3.17	3.31	3.326	3.327	3.33	3.374	3.437	3.54	3.7	3.8
		MoS_2	WS_2	VS_2	VSe_2	$MoSe_2$	WSe_2	NbS_2	TiS_2	$NbSe_2$	$TiSe_2$	SnS_2	$SnSe_2$
5.714	BiS	1.81	1.81	1.80	1.73	1.72	1.72	1.72	1.69	1.66	1.61	1.54	1.50
5.852	SnS	1.86	1.86	1.85	1.77	1.76	1.76	1.76	1.73	1.70	1.65	1.58	1.54
5.875	LaS	1.87	1.87	1.85	1.78	1.77	1.77	1.76	1.74	1.71	1.66	1.59	1.54
5.927	SnSe	1.88	1.88	1.87	1.79	1.78	1.78	1.78	1.76	1.72	1.67	1.60	1.56
6	LaSe	1.91	1.90	1.89	1.81	1.80	1.80	1.80	1.78	1.75	1.69	1.62	1.57
6	PbS	1.90	1.90	1.89	1.81	1.80	1.80	1.80	1.78	1.75	1.69	1.62	1.57
6.026	BiSe	1.91	1.91	1.90	1.82	1.81	1.81	1.81	1.79	1.75	1.70	1.63	1.58
6.028	PbSe	1.91	1.91	1.90	1.82	1.81	1.81	1.81	1.79	1.75	1.70	1.63	1.58

Figure S1: Exact mismatch ratios a_2/a_1. Lattice parameters of the considered compounds are experimental values.

a_2 (Å) \ a_1 (Å)		3.15	3.15	3.17	3.31	3.326	3.327	3.33	3.374	3.437	3.54	3.7	3.8
		MoS_2	WS_2	VS_2	VSe_2	$MoSe_2$	WSe_2	NbS_2	TiS_2	$NbSe_2$	$TiSe_2$	SnS_2	$SnSe_2$
5.714	BiS	1.10	1.10	1.11	1.16	1.16	1.16	1.17	1.18	1.20	1.24	1.30	1.33
5.852	SnS	1.08	1.08	1.08	1.14	1.14	1.14	1.14	1.15	1.18	1.21	1.26	1.30
5.875	LaS	1.07	1.07	1.08	1.13	1.13	1.13	1.13	1.15	1.17	1.21	1.26	1.30
5.927	SnSe	1.06	1.06	1.07	1.12	1.13	1.12	1.12	1.14	1.16	1.20	1.25	1.29
6	LaSe	1.05	1.05	1.06	1.10	1.11	1.11	1.11	1.13	1.15	1.18	1.23	1.27
6	PbS	1.05	1.05	1.06	1.10	1.11	1.11	1.11	1.13	1.15	1.18	1.23	1.27
6.026	BiSe	1.05	1.05	1.05	1.10	1.10	1.10	1.11	1.12	1.14	1.18	1.23	1.27
6.028	PbSe	1.05	1.05	1.05	1.10	1.10	1.10	1.11	1.12	1.14	1.18	1.23	1.26

Figure S2: Exact misfits stechiometry $1+\delta = 2 \times (a_1/a_2)$. Lattice parameters of the considered compounds are experimental values.

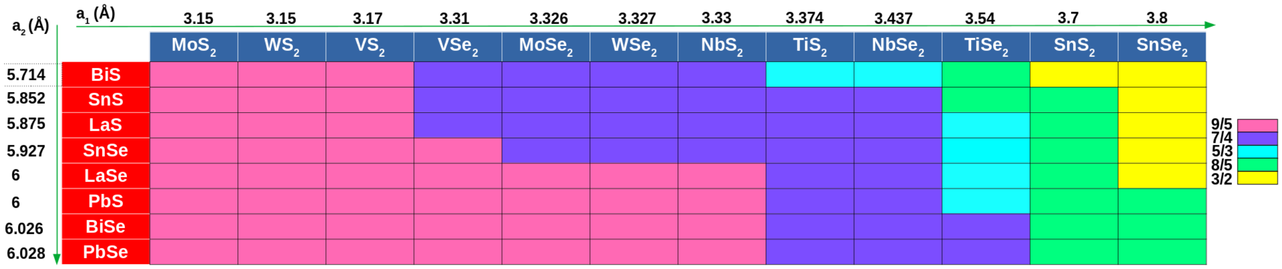

Figure S3: Table of the periodic approximant extracted from the rocksalt and TMDs lattice mismatch. Lattice parameters of the considered compounds are experimental values.

mismatch direction are slightly strained in order to build a commensurate misfit structure. We obtain a_{TiSe_2}=3.6Å (tensile strain of ≈ 2%, exp. value a_{TiSe_2}=3.54Å) and a_{SnSe_2}=3.8Å (compressive strain of ≈ 0.3%, exp. value a_{SnSe_2}=3.81Å), respectively. In the other in-plane

direction, we find b_{TiSe_2}=6.0191Å and b_{SnSe_2}=6.5818Å, respectively. Starting from this cell, a supercell is built according to the misfit proportions (5×1 and a 3×1 supercell for the cases of $TiSe_2$/LaSe and $SnSe_2$/LaSe misfits, respectively). Regarding the Q-layer rocksalt, we optimized LaSe with centered orthorhombic cell and slightly strained in-plane lattice parameter in order to obtain some commensurability with the considered TMD. A 3×1 LaSe supercell with in-plane lattice parameters $a_{LaSe} \approx b_{LaSe}$=6Å (tensile strain of $\approx$ 0.5%, exp. value $a_{LaSe} \approx b_{LaSe}$=5.97Å) is considered in order to match with $TiSe_2$, and a $2 \times 1 \times 1$ with lattice parameter a_{LaSe}=5.7Å (compressive strain of $\approx$ 4%, exp. value a_{LaSe}=5.97Å) and b_{LaSe}=6.5818 Å (tensile strain of $\approx$ 10%, exp. value a_{LaSe}=5.97Å) is considered in order to match with $SnSe_2$. The two cells (one for the TMD and one for the rocksalt) are then appropriately assembled to build the slab system, which possesses a P1 symmetry in the most general case. This is composed of a Q-layer LaSe sandwiched between two $TiSe_2$ (or $SnSe_2$) single layers. The final $(LaSe)_{1.18}(TiSe_2)_2$ cell is composed of 84 atoms, the mismatch ratio is a_2/a_1 =(6Å)/(3.6Å) = 1.66 $\simeq$ 5/3, leading to lattice parameter of the misfit supercell equal to a = 18Å. Instead, $(LaSe)_{1.27}(SnSe_2)_2$ is composed of 52 atoms, the mismatch ratio is a_2/a_1 =(5.7Å)/(3.8Å) = 1.57 $\simeq$ 3/2, leading to lattice parameter of the misfit supercell equal to a = 11.4Å. In the tables S1 and S2 we report the optimized atomic positions expressed in crystalline coordinates. The crystal cell of $(LaSe)_{1.18}(TiSe_2)_2$ is orthorhombic with a=18Å, b=6.0191Å, c=30Å. The crystal cell of $(LaSe)_{1.27}(SnSe_2)_2$ is orthorhombic with a=11.4Å, b=6.581789Å, c=30Å.

II. Technical details

For what concerns the convergence parameters of DFT calculations of surface of the misfits, we employ a $2 \times 8 \times 1$ a Monkhorst-Pack k-points grid and a Gaussian smearing of 0.025 Ry for Brillouin Zone (BZ) sampling. We use the generalized gradient approximation in the Perdew–Burke–Ernzerhof[1] parametrization for the exchange-correlation functional.

In the calculation of surface properties, as the interaction among transition metal dichalcogenides layers is missing and only covalent bonds among the rocksalt and the transition metal dichalcogenides are present, we did not consider any Van der Waals correction. For the case of the bulk superconducting properties of $(LaSe)_{1.27}(SnSe_2)_2$, where the interaction among adjacent $SnSe_2$ layers is present, we included the Van der Waals corrections Grimme-D3[2] in the cell relaxation in order to carefully reproduce the interlayer distance.

Spin orbit coupling (SOC) is included in all the electronic structure calculations in the main text. We verified that the inclusion of SOC marginally modifies the band structure of misfit layer compounds containing Bi and Pb rocksalts. Relativistic effects are negligible for the electronic properties of $(La_xPb_{1-x}Se)_{1.18}(TiSe_2)_2$ family of compounds. For this reason, SOC is neglected in the calculations made for these compounds in the SI.

In case of $TiSe_2$ componunds, we employ PBE+U method described in Refs.[3,4] in order to take into account the strong correlation effects due to the localized d orbitals of Ti. The Hubbard correction is set to U= 3.25 eV, consistently with previous work on bulk $TiSe_2$ where a good agreement with ARPES spectra was demonstrated.[5]

We consider the following pseudopotential configurations taken from the Vanderbilt[6] and PSlibrary[7] distributions. (i) For Se, norm-conserving pseudopotentials. (ii) For Ti, ultrasoft pseudopotentials from Vanderbilt distribution. (iii) For La, ultrasoft pseudopotentials from the Vanderbilt distributions. (iv) For Sn, Optimized Norm-Conserving Vanderbilt Pseudopotential. (v) For Pb, ultrasoft pseudopotentials from the PSlibrary distributions. The values of kinetic energy cutoff for plane-wave basis set is set to 50 Ry and for charge density 600 Ry respectively for all the misfits. All systems contain a total of 15Å of vacuum between the periodic images. The atomic position of the slab are relaxed, by means of the Broyden-Fletcher-Goldfarb-Shanno (BFGS) algorithm, with a convergence threshold of 10^{-4} Ry on the total energy difference between consecutive structural optimisation steps and of 10^{-3} Ry/Bohr on all forces components.

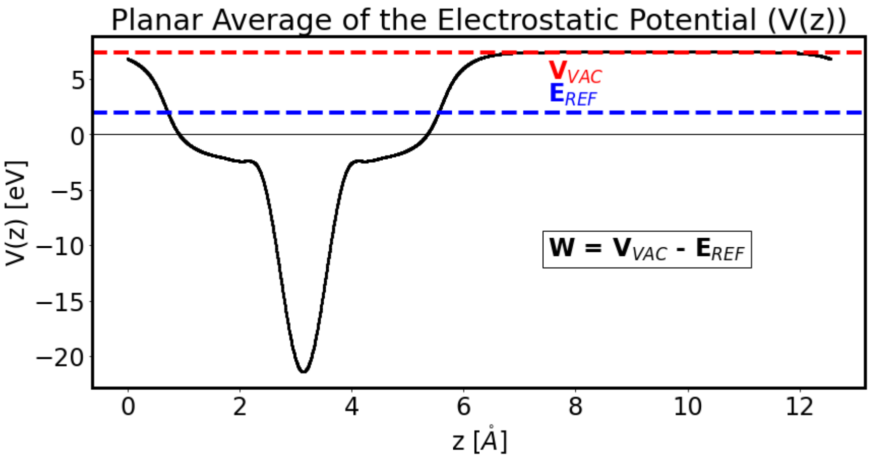

Figure S4: The calculation of V(z), which is the planar averaged electrostatic potential along the stacking axis z, is depicted in this figure. The negative peaks represents the spacial region in which the material is located, then we can determine the value of $V_{\rm VAC}$ in the vacuum region of the plot (red line). Then, the work function is $W = V_{\rm VAC} - E_{\rm Ref}$ where for every material $E_{\rm Ref}$ corresponds to E_F (E_{VBM}) for metals (insulators).

III. Band Alignment Calculation

We use the band alignment method in order to make a computational screening of MLCs heterostructures from their elemental constituents. The work function is the amount of energy needed to extract an electron from a solid so that it exits with zero kinetic energy. Therefore, the difference of work functions of two compounds A and B is defined as:

$$\Delta W = W_A - W_B = V_{\rm VAC} - {E^A}_{\rm Ref} - V_{\rm VAC} + {E^B}_{\rm Ref} = {E^B}_{\rm Ref} - {E^A}_{\rm Ref} = -\Delta E_{\rm Ref} \qquad (1)$$

Thus, if $\Delta W > 0$ then ${E^B}_{\rm Ref} > {E^A}_{\rm Ref}$ and electrons will flow from the material B to the material A. In this context, the estimation of W leads to the knowledge of charge transfer mechanism in heterostructures by means of the band alignment method.[8,9] We calculate the work functions (W) of isolated subsystems, namely Q-layer rocksalts and single layer TMDs. The procedure depicted in Fig. S4 is the following: we estimate for each system the energy reference $E_{\rm Ref}$ namely Fermi energy for metals (E_F) or valence band maximum for insulators

and semiconductors (VBM). Then we define the work function as $W = V_{\rm VAC} - E_{\rm Ref}$ where

$$V_{\rm VAC} = V_{\rm VAC}(z) = \frac{1}{A}\int_A dxdy\ V_{\rm VAC}(x, y, z) \qquad (2)$$

is the value of the electrostatic potential along z averaged in the plane (A being the surface area in the plane of the unit cell) in the vacuum region. Numerical values of work functions are calculated in the Perdew-Burke-Ernzerhof (PBE) scheme[1] with the QE package.[10] We employ pseudopotentials (pseudo efficiency) each with a proper converged kinetic energy cutoff for plane-wave basis set. The Brillouin zone integration is performed with a Monkhorst-Pack grid[11] of 21 $\times$ 21 $\times$ 1 k-points and using a Gaussian broadening of 0.01 Ry, proven to be sufficient to accurately determine the electronic properties of all systems under consideration. The in-plane lattice parameter of all the considered structures is fixed as the experimental one of each subsystem, namely the Q-layer rocksalts and the monolayer TMDs (see Fig. S1). The internal coordinates are fully optimised by means of the Broyden-Fletcher-Goldfarb-Shanno (BFGS) algorithm, with a convergence threshold of 10^{-4} Ry on the total energy difference between consecutive structural optimisation steps and of 10^{-3} Ry/Bohr on all forces components.

IV. Band unfolding method applied to $(La_xPb_{1-x}Se)_{1.18}(TiSe_2)_2$

We use unfolding method based of effective band structure (EBS)[12] as implemented in the BandsUP software.[13] We report the full calculation of $(LaSe)_{1.18}(TiSe_2)_2$ with partial substitution of Pb atoms ($(La_xPb_{1-x}Se)_{1.18}(TiSe_2)_2$ with $x = 1.0, 0.834, 0.67, 0.5, 0.34, 0.167, 0.0$). In Fig. S5 we can see the band unfolding in the PBE+U scheme of misfit SC $(La_xPb_{1-x}Se)_{1.18}(TiSe_2)_2$ along the Γ-M-K-Γ path of the PC single layer $TiSe_2$ IBZ. The colormap represent the unfolded band structure with different percentage of Pb atoms, over which we superimpose (in red) the band structure of the PC single layer $TiSe_2$. We can clearly see that the single layer $TiSe_2$ band structure is preserved, which is a sign of rigid doping mechanism in the

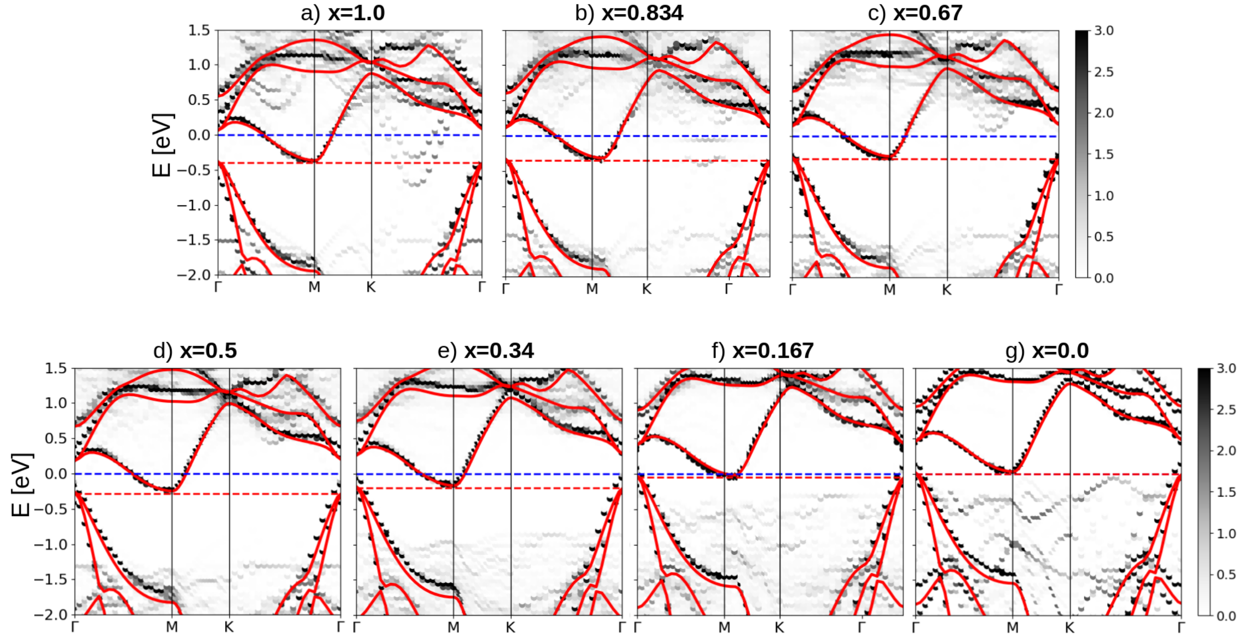

Figure S5: Band unfolding onto the hexagonal Brillouin Zone (BZ) of a single layer $TiSe_2$ in the PBE+U scheme of misfit supercell $(La_xPb_{1-x}Se)_{1.18}(TiSe_2)_2$, with $x =$ (a) 1.0, (b) 0.834, (c) 0.67, (d) 0.5, (e) 0.34, (f) 0.167, (g) 0.0. Darker regions in the colormap represent the most relevant eigenvalues of the misfit band structure along the $TiSe_2$ first BZ. In red isolated single layer $TiSe_2$ band structure is superimposed onto the unfolded one. Blue (red) dashed line corresponds to the Fermi level E_F of the misfit (single layer $TiSe_2$).

misfit. The Fermi level of the TMD in the misfit (blue) is shifted with respect to the single layer one (red) of an amount which is directly related to the presence of Pb in the rocksalt. Indeed, it decreases as the number of Pb atoms increases, leading to a perfect superposition of Fermi level (almost zero doping) with full lead substitution. The key concept behind this behaviour is detailed in the main text and is based on work functions determination. In Fig. S6 we also show the same calculations of $(La_xPb_{1-x}Se)_{1.18}(TiSe_2)_2$ in the PBE scheme for $x = 1.0, 0.67, 0.5, 0.34, 0.0$, without taking into account the Hubbard correction. The main difference between these two approaches resides in the energy dispersion around the Fermi level, especially the overlap/gap between the Se-4p valence band in Γ and the Ti-3d conduction band in M. Indeed, as shown in Ref.[5] the introduction of Hubbard interaction leds to a better comparison with ARPES experiments in which shows that monolayer $1TTiSe_2$ is a perfectly compensated semimetal.

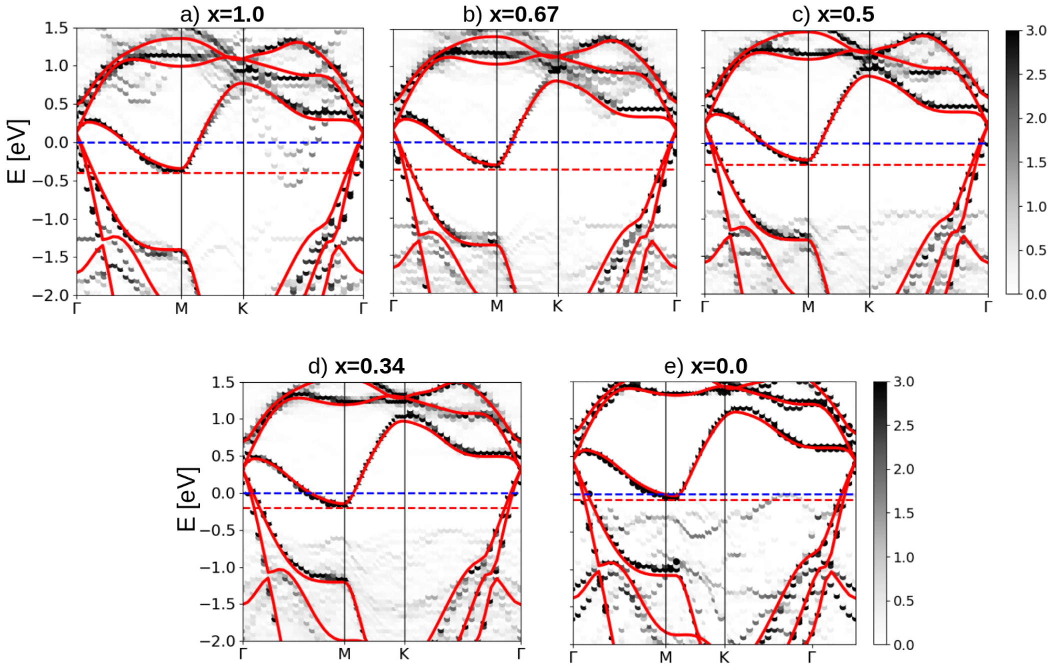

Figure S6: Band unfolding in the PBE scheme of misfit supercell $(La_xPb_{1-x}Se)_{1.18}(TiSe_2)_2$, with x = (a) 1.0, (b) 0.67, (c) 0.5, (d) 0.34, (e) 0.0, onto the hexagonal BZ of a single layer $TiSe_2$. Darker regions in the colormap represent the most relevant eigenvalues of the misfit band structure along the $TiSe_2$ IBZ.In red isolated single layer $TiSe_2$ band structure is superimposed onto the unfolded one. Blue (red) dashed line corresponds to the Fermi level E_F of the misfit (single layer $TiSe_2$).

V. Doping-induced Superconductivity

As detailed in the text, in order to recover the $(LaSe)_{1.27}(SnSe_2)_2$ surface and bulk behaviour, we used the 2D material FET setup[14] to precisely dope $SnSe_2$ as it is inside the misfit. A Coulomb long range interaction cutoff is placed at $z_{cut} = c/2$ with c being the unit-cell size in the direction perpendicular to the 2D plane: c is set opportunely for single and double layer $SnSe_2$ at 16.14Å and 25.83Å, respectively. For $SnSe_2$ monolayer we use a single gate setup, placing charged plate modelling a single gate electrode at $z_{bot} = -0.25c$ with a charge of +0.7, equal and opposite to the one of the single layer $SnSe_2$ to ensure charge neutrality. For $SnSe_2$ bilayer we use a double gate configuration, with the bilayer sandwiched between two charged plates at $z_{bot} = -0.266c$ and $z_{top} = +0.266c$ each with a charge of ρ=+(-)0.7, such that $\rho_{tot} = \rho_{2L} + \rho_{bot} + \rho_{top} = 0$. For all systems, potential barriers V with a height

of $V_H = 2.5$ Ry are placed before the gates at $z_V = z_{bot} + 0.1$ ($z_V = z_{top} - 0.1$) in order to prevent the ions from moving too close to the gate electrodes.

The harmonic phonon frequencies are evaluated within density-functional perturbation theory[15] on a 7×7×1 phonon momentum grid (**q**-grid) and 21×21×1 electron-momentum grid (**k**-grid). The slightly imaginary acoustic phonons (order of 8 cm^{-1}) observable in the phonon dispersion are an artifact linked to the acoustic sum rule breaking in linear response. To avoid spurious effects, we did not impose the acoustic sum rule manually since it has been shown that in field-effect gated 2D system one of the three acoustic modes remains finite at Γ (see[14]). The correct evaluation of the electron-phonon coupling properties of doped $SnSe_2$ requires a precise knowledge of electron-phonon matrix elements for very dense electron and phonon momentum grids. Since the direct calculation of electron-phonon matrix elements over a ultradense **q**- and **k**-point grids is very time consuming in linear response, we perform a Wannier interpolation of the electron-phonon coupling as described in Ref.[16] We used the Wannier90[17] code to obtain the Bloch to Wannier transformation. We use as starting guess of the Maximally Localized Wannier Function procedure three *p*-like orbitals at every chalcogen site and 5 *d*-like orbitals at any molybdenum site. Within this approach, the electron-phonon matrix elements are first calculated on a coarse 7×7×1 **q** grid and 21×21×1 **k**-grid, and then Wannier interpolated to 96×96×1 **q**- and **k**- grids in order to evaluate the electron-phonon coupling parameter λ and the isotropic Eliashberg function $\alpha^2 F(\omega)$. We employ a Gaussian smearing of 0.001 Ry for **k**- and **q**- summations in λ.

The superconducting gap is evaluated by solving the Migdal-Eliashberg equations[18]in the Wannier basis over the imaginary frequency axis and then by performing analytic continuation to the real axis using N-point Padé approximants.[19] In order to perform the **k**- and $\mathbf{k} + \mathbf{q}$- summations to solve the Migdal-Eliashberg equations on the imaginary axis, we generate random electron momenta on the Brillouin zone, and select 2048 of them having at least one eigenvalue within 0.2 eV of the Fermi surface. The Matsubara summation was truncated at 128 frequencies, where convergence is reached. The superconducting critical

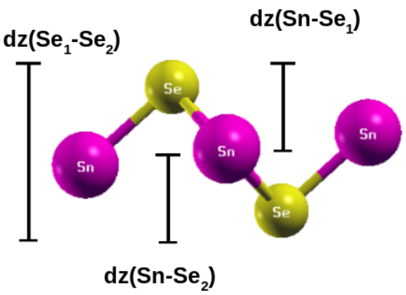

dz (Å)	Misfit	1L-FET	1L
dz(Se$_1$-Se$_2$)	3.34	3.37	3.19
dz(Sn-Se$_1$)	1.78	1.79	1.59
dz(Sn-Se$_2$)	1.56	1.58	1.59

Figure S7: Intralayer spacing (d_z) between Sn and Se in $SnSe_2$. Comparison between values of d_z in isolated monolayer (1L), monolayer in the MLC $(LaSe)_{1.27}(SnSe_2)_2$ (misfit), and single-gate doped monolayer (1L-FET) with 0.7 electron/Sn. The charge transfer exerted by LaSe in $(LaSe)_{1.27}(SnSe_2)_2$ modifies out-of-plane spacing (d_z) in monolayer $SnSe_2$. The structural optimization in a FET setup recover this geometrical behaviour.

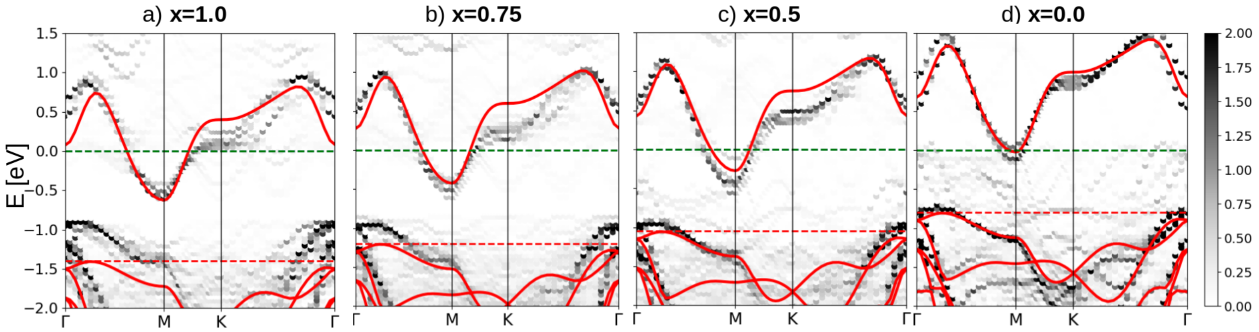

Figure S8: Band unfolding of misfit supercell $(La_xPb_{1-x}Se)_{1.27}(SnSe_2)_2$, with $x =$ (a) 1.0, (b) 0.75, (c) 0.5 onto the hexagonal BZ of a single layer $TiSe_2$. Darker regions in the colormap represent the most relevant eigenvalues of the misfit band structure along the monolayer $SnSe_2$ IBZ. In red solid line, the superimposed band structure of an isolated single layer $SnSe_2$ is shown.

temperature is then evaluated by determining the temperature where the superconducting gap becomes zero. We use a Morel-Anderson pseudopotential[20] $\mu^* = 0.1$ to parameterize the Coulomb repulsion in the superconducting state. The Wannier interpolation of the electron-phonon matrix elements, as well as the solution of the Migdal-Eliashberg equations have been performed within EPIq (Electron-Phonon Interpolation over **q**- and **k**-points), an open-source in-house software.[21]

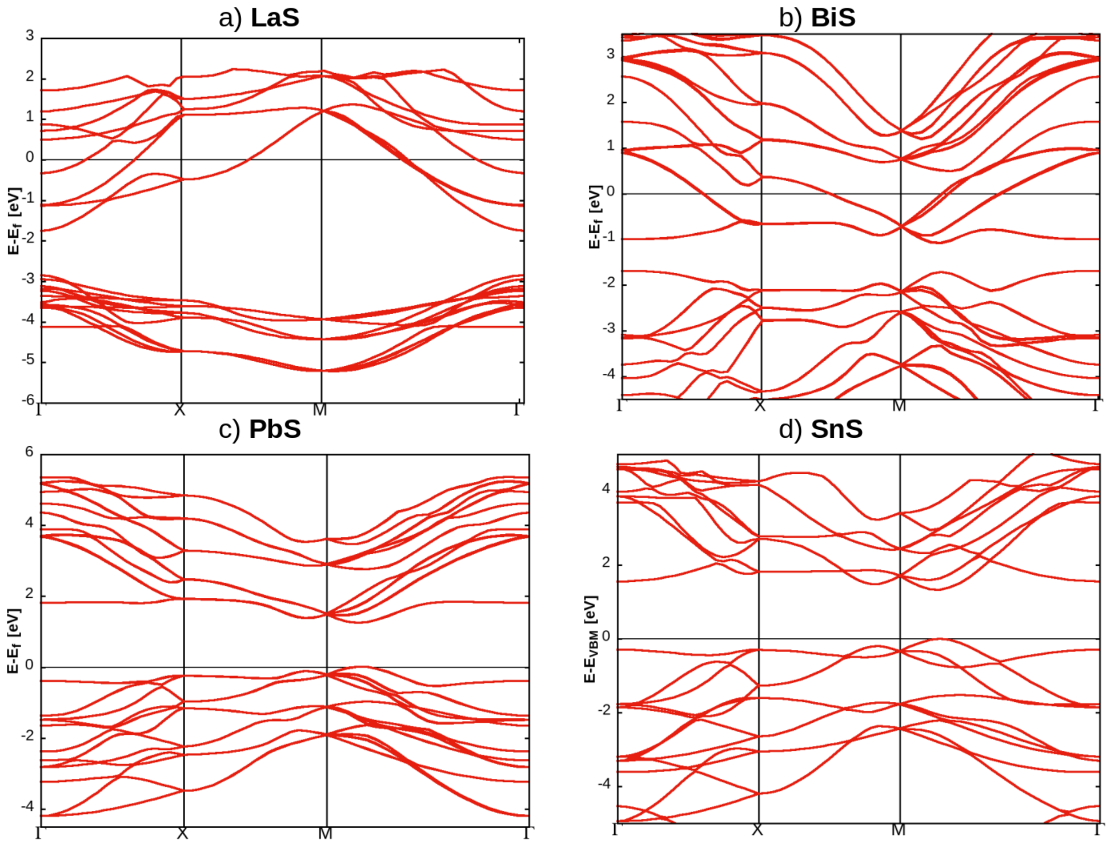

Figure S9: Calculated band structure of isolated sulfide rocksalt bilayers. a) LaS, b) BiS, c) PbS, d) SnS. Bands are shifted with respect to Fermi level for metals or valence band maximum for insulators. Spin orbit coupling is included in Bi and Pb compound calculations.

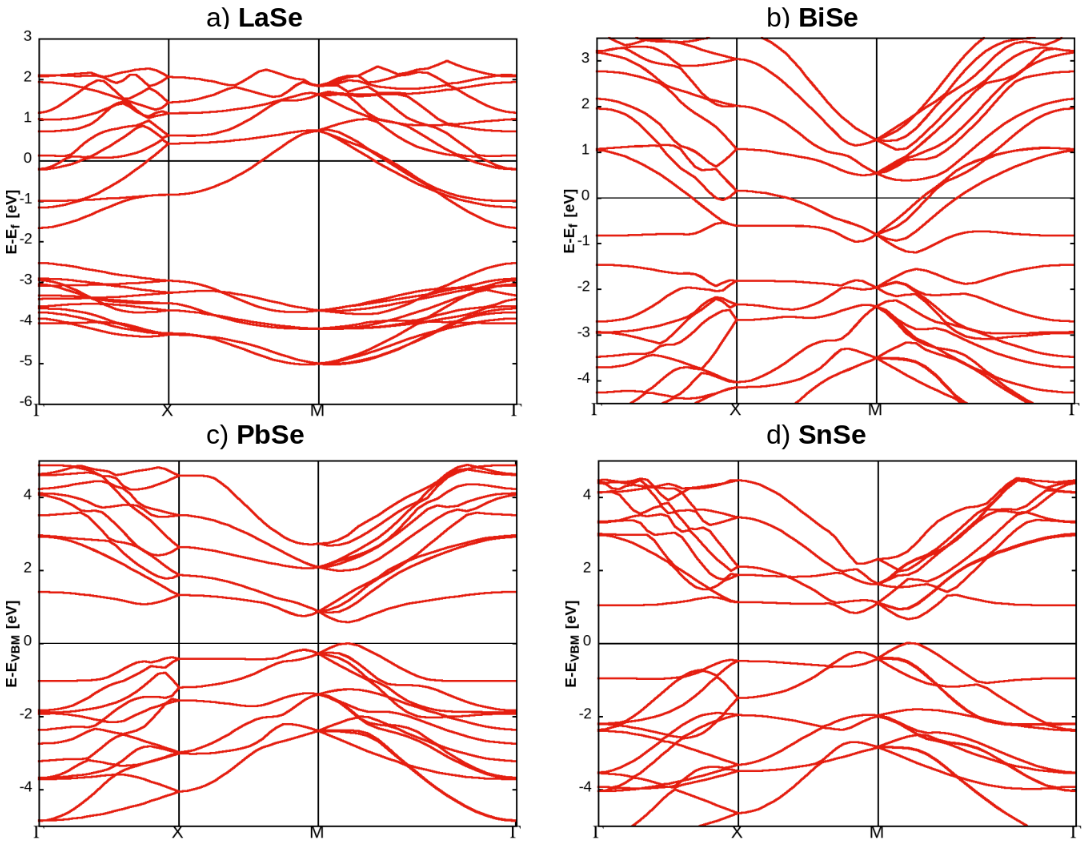

Figure S10: Calculated band structure of isolated selenide rocksalt bilayers. a) LaSe, b) BiSe, c) PbSe, d) SnSe. Bands are shfted with respect to Fermi level for metals or valence band maximum for insulators. Spin orbit coupling is included in Bi and Pb compound calculations.

Table S1: Optimized atomic positions of MLC $(LaSe)_{1.18}(TiSe_2)_2$ in crystal coordinates.

Ti -0.0054 -0.0731 -0.0370	Se 0.8977 0.7585 0.0188	Se 0.0936 0.2510 0.3084
Se -0.0062 0.2531 0.0162	Se 0.7942 0.5854 -0.0859	Ti 0.0939 0.5882 0.3624
Se 0.0953 0.0879 -0.0863	Se 0.0008 0.0174 0.1243	Se 0.0927 0.9256 0.4139
Ti 0.0943 0.4255 -0.0349	La 0.1680 -0.008 0.1007	Se -0.0067 0.7526 0.3108
Se 0.0924 0.7604 0.0193	Se 0.16712 0.4987 0.1246	Ti 0.1932 0.0858 0.3642
Se -0.0054 0.5864 -0.0862	La 0.0006 0.5253 0.0992	Se 0.1933 0.4260 0.4138
Ti 0.1943 -0.0774 -0.0354	Se 0.3343 0.0025 0.1241	Se 0.2898 0.2565 0.3094
Se 0.1955 0.2608 0.0176	La 0.5005 0.0254 0.0991	Ti 0.2935 0.5859 0.3634
Se 0.2941 0.0858 -0.0858	Se 0.5007 0.5173 0.1243	Se 0.2942 0.9253 0.4136
Ti 0.2944 0.4233 -0.0350	La 0.3333 0.4986 0.1011	Se 0.1956 0.7587 0.3107
Se 0.2929 0.7616 0.0183	Se 0.6671 -0.0013 0.1245	Ti 0.3929 0.0888 0.3619
Se 0.1948 0.5849 -0.0858	La 0.8333 -0.0015 0.1009	Se 0.3939 0.4256 0.4134
Ti 0.3943 -0.0740 -0.0351	Se 0.8343 0.5024 0.1240	Se 0.4933 0.2523 0.3107
Se 0.3975 0.2589 0.0190	La 0.6681 0.4920 0.1007	Ti 0.4934 0.5909 0.3633
Se 0.4944 0.0863 -0.0862	Se 0.0012 0.5179 0.2029	Se 0.4931 0.9289 0.4132
Ti 0.4944 0.4272 -0.0370	La 0.1681 0.4936 0.2276	Se 0.3945 0.7502 0.3083
Se 0.4938 0.7533 0.0162	Se 0.1675 -0.0010 0.2035	Ti 0.5940 0.0880 0.3623
Se 0.3935 0.5879 -0.0861	La 0.0006 0.0269 0.2278	Se 0.5927 0.4255 0.4138
Ti 0.5943 -0.0745 -0.0348	Se 0.3343 0.5035 0.2035	Se 0.6958 0.2585 0.3106
Se 0.5924 0.2606 0.0193	La 0.5007 0.5268 0.2277	Ti 0.6934 0.5858 0.3642
Se 0.6948 0.0846 -0.0858	Se 0.5012 0.0177 0.2029	Se 0.6933 0.9261 0.4138
Ti 0.6946 0.4227 -0.0354	La 0.3346 0.0022 0.2266	Se 0.5936 0.7508 0.3083
Se 0.6957 0.7609 0.0176	Se 0.6675 0.4989 0.2034	Ti 0.7937 0.0861 0.3634
Se 0.5952 0.5878 -0.0862	La 0.8345 0.5024 0.2266	Se 0.7942 0.4255 0.4136
Ti 0.7946 -0.0768 -0.0351	Se 0.8343 0.0038 0.2034	Se 0.8946 0.2505 0.3084
Se 0.7931 0.2616 0.0182	La 0.6680 -0.0064 0.2276	Ti 0.8931 0.5889 0.3621
Se 0.8938 0.0875 -0.0862	Ti -0.0066 0.0913 0.3634	Se 0.8940 0.9258 0.4135
Ti 0.8946 0.4259 -0.0353	Se -0.0069 0.4292 0.4134	Se 0.7810 0.7567 0.3095

Table S2: Optimized atomic positions of MLC $(LaSe)_{1.27}(SnSe_2)_2$ in crystal coordinates.

Sn -0.0351 -0.0840 -0.0357	Se 0.0364 -0.0018 0.1242	Se 0.1370 0.2524 0.3042
Se -0.0403 0.2573 0.0235	La 0.2870 -0.0254 0.0954	Sn 0.1333 0.5963 0.3634
Se 0.1216 0.0782 -0.0947	Se 0.2839 0.5087 0.1235	Se 0.1441 0.9265 0.4183
Sn 0.1331 0.4172 -0.0421	La 0.0308 0.5327 0.1003	Se -0.0430 0.7643 0.3078
Se 0.1383 0.7529 0.0194	Se 0.5356 0.0040 0.1241	Sn 0.3008 0.0972 0.3621
Se -0.0430 0.5890 -0.0900	La 0.7867 0.0496 0.1001	Se 0.3022 0.4218 0.4157
Sn 0.2993 -0.0781 -0.0471	Se 0.7842 0.5003 0.1287	Se 0.4616 0.2554 0.3039
Se 0.3008 0.2404 0.0152	La 0.5358 0.5109 0.1004	Sn 0.4669 0.5965 0.3630
Se 0.4745 0.0780 -0.0960	Se 0.0358 0.5067 0.2033	Se 0.4593 0.9234 0.4173
Sn 0.4640 0.4188 -0.0440	La 0.2871 0.4629 0.2272	Se 0.3064 0.7584 0.3033
Se 0.45514 0.7487 0.0194	Se 0.2839 0.0119 0.1986	Sn 0.6352 0.0954 0.3693
Se 0.2969 0.5791 -0.0979	La 0.0353 0.0047 0.2270	Se 0.6237 0.4342 0.4219
Sn 0.6313 -0.0837 -0.0361	Se 0.5367 0.5135 0.2031	Se 0.8032 0.2718 0.3120
Se 0.6350 0.2510 0.0232	La 0.7873 0.5378 0.2319	Sn 0.8014 0.5906 0.3743
Se 0.8000 0.0901 -0.0883	Se 0.7835 0.0035 0.2038	Se 0.7987 0.9334 0.4251
Sn 0.7987 0.4154 -0.0346	La 0.5304 -0.0218 0.2271	Se 0.6310 0.7592 0.3080
Se 0.8035 0.7543 0.0239	Sn -0.0339 0.0936 0.3714	
Se 0.6421 0.5864 -0.0909	Se -0.0236 0.4345 0.4233	

References

(1) Perdew, J. P.; Burke, K.; Ernzerhof, M. Generalized Gradient Approximation Made Simple. *Physical Review Letters* **1996**, *77*, 3865–3868, DOI: 10.1103/physrevlett.77.3865.

(2) Grimme, S.; Ehrlich, S.; Goerigk, L. Effect of the damping function in dispersion corrected density functional theory. *Journal of Computational Chemistry* **2011**, *32*, 1456–1465, DOI: https://doi.org/10.1002/jcc.21759.

(3) Cococcioni, M.; de Gironcoli, S. Linear response approach to the calculation of the effective interaction parameters in the LDA + U method. *Phys. Rev. B* **2005**, *71*, 035105, DOI: 10.1103/PhysRevB.71.035105.

(4) Himmetoglu, B.; Floris, A.; de Gironcoli, S.; Cococcioni, M. Hubbard-corrected DFT energy functionals: The LDA+U description of correlated systems. *International Journal of Quantum Chemistry* **2014**, *114*, 14–49, DOI: https://doi.org/10.1002/qua.24521.

(5) Bianco, R.; Calandra, M.; Mauri, F. Electronic and vibrational properties of $TiSe_2$ in the charge-density-wave phase from first principles. *Phys. Rev. B* **2015**, *92*, 094107, DOI: 10.1103/PhysRevB.92.094107.

(6) Vanderbilt, D. Soft self-consistent pseudopotentials in a generalized eigenvalue formalism. *Phys. Rev. B* **1990**, *41*, 7892–7895, DOI: 10.1103/PhysRevB.41.7892.

(7) Dal Corso, A. Pseudopotentials periodic table: From H to Pu. *Computational Materials Science* **2014**, *95*, 337–350, DOI: https://doi.org/10.1016/j.commatsci.2014.07.043.

(8) Fall, C. J.; Binggeli, N.; Baldereschi, A. Deriving accurate work functions from thin-slab calculations. **1999**, *11*, 2689, DOI: 10.1088/0953-8984/11/13/006.

(9) Stengel, M.; Aguado-Puente, P.; Spaldin, N. A.; Junquera, J. Band alignment at metal/ferroelectric interfaces: Insights and artifacts from first principles. *Phys. Rev. B* **2011**, *83*, 235112, DOI: 10.1103/PhysRevB.83.235112.

(10) Giannozzi, P.; Baseggio, O.; Bonfà, P.; Brunato, D.; Car, R.; Carnimeo, I.; Cavazzoni, C.; de Gironcoli, S.; Delugas, P.; Ferrari Ruffino, F.; Ferretti, A.; Marzari, N.; Timrov, I.; Urru, A.; Baroni, S. Quantum ESPRESSO toward the exascale. *The Journal of Chemical Physics* **2020**, *152*, 154105.

(11) Monkhorst, H. J.; Pack, J. D. Special points for Brillouin-zone integrations. *Phys. Rev. B* **1976**, *13*, 5188–5192, DOI: 10.1103/PhysRevB.13.5188.

(12) Popescu, V.; Zunger, A. Extracting E versus $\vec{k}$ effective band structure from supercell calculations on alloys and impurities. *Phys. Rev. B* **2012**, *85*, 085201, DOI: 10.1103/PhysRevB.85.085201.

(13) Medeiros, P. V. C.; Stafström, S.; Björk, J. Effects of extrinsic and intrinsic perturbations on the electronic structure of graphene: Retaining an effective primitive cell band structure by band unfolding. *Phys. Rev. B* **2014**, *89*, 041407, DOI: 10.1103/PhysRevB.89.041407.

(14) Sohier, T.; Calandra, M.; Mauri, F. Density functional perturbation theory for gated two-dimensional heterostructures: Theoretical developments and application to flexural phonons in graphene. *Phys. Rev. B* **2017**, *96*, 075448, DOI: 10.1103/PhysRevB.96.075448.

(15) Baroni, S.; de Gironcoli, S.; Dal Corso, A.; Giannozzi, P. Phonons and related crystal properties from density-functional perturbation theory. *Rev. Mod. Phys.* **2001**, *73*, 515–562, DOI: 10.1103/RevModPhys.73.515.

(16) Calandra, M.; Profeta, G.; Mauri, F. Adiabatic and nonadiabatic phonon dis-

persion in a Wannier function approach. *Phys. Rev. B* **2010**, *82*, 165111, DOI: 10.1103/PhysRevB.82.165111.

(17) Pizzi, G. et al. Wannier90 as a community code: new features and applications. *Journal of Physics: Condensed Matter* **2020**, *32*, 165902, DOI: 10.1088/1361-648x/ab51ff.

(18) Allen, P. B.; Mitrović, B. In *Theory of Superconducting Tc*; Ehrenreich, H., Seitz, F., Turnbull, D., Eds.; Solid State Physics; Academic Press, 1983; Vol. 37; pp 1–92, DOI: https://doi.org/10.1016/S0081-1947(08)60665-7.

(19) Vidberg, H. J.; Serene, J. W. Solving the Eliashberg equations by means of N-point Padé approximants. *Journal of Low Temperature Physics* **1977**, *29*, 179–192, DOI: 10.1007/BF00655090.

(20) Morel, P.; Anderson, P. W. Calculation of the Superconducting State Parameters with Retarded Electron-Phonon Interaction. *Phys. Rev.* **1962**, *125*, 1263–1271, DOI: 10.1103/PhysRev.125.1263.

(21) Marini, G.; Marchese, G.; Profeta, G.; Sjakste, J.; Macheda, F.; Vast, N.; Mauri, F.; Calandra, M. EPIq : an open-source software for the calculation of electron-phonon interaction related properties. **2023**, DOI: https://doi.org/10.48550/arXiv.2306.15462.
